# Sodium Alginate Prevents Non-Alcoholic Fatty Liver Disease by Modulating the Gut–Liver Axis in High-Fat Diet-Fed Rats

**DOI:** 10.3390/nu14224846

**Published:** 2022-11-16

**Authors:** Hui Zhao, Xiang Gao, Zhizuo Liu, Lei Zhang, Xuan Fang, Jianping Sun, Zhaofeng Zhang, Yongye Sun

**Affiliations:** 1 Department of Nutrition and Food Hygiene, College of Public Health, Qingdao University, Qingdao 266071, China; 2 College of Life Sciences, Qingdao University, Qingdao 266071, China; 3 Women and Children’s Hospital Affiliated to Qingdao University, Qingdao 266071, China; 4 Qingdao Institute for Food and Drug Control, Qingdao 266071, China; 5 Qingdao Centers for Disease Control and Prevention, Qingdao 266033, China; 6 Department of Nutrition and Food Hygiene, School of Public Health, Peking University Health Science Center, Beijing 100191, China; 7 Key Laboratory of Food Safety Toxicology Research and Evaluation, Beijing 100191, China

**Keywords:** non-alcoholic fatty liver disease, sodium alginate, intestinal flora, metabolites, inflammation, high-fat diet, serum lipids

## Abstract

Previous studies have suggested that the sodium alginate (SA) is beneficial for the treatment of non-alcoholic fatty liver disease (NAFLD), while the potential mechanisms are largely unknown. The present study aimed to clarify the effects and potential mechanisms of SA in preventing NAFLD via the gut−liver axis. Thirty-two male Sprague−Dawley rats were randomly divided into four groups: normal control group (NC); high-fat diet group (HFD); HFD with 50 mg/kg/d sodium alginate group (LSA); HFD with 150 mg/kg/d sodium alginate group (HSA). After 16 weeks, the rats were scarified to collect blood and tissues. The results indicated that SA significantly reduced their body weight, hepatic steatosis, serum triglyceride (TG), alanine transaminase (ALT) and tumor necrosis factor α (TNF-α) levels and increased serum high-density lipoprotein-cholesterol (HDL-C) levels in comparison with HFD group (*p* < 0.05). The elevated mRNA and protein expression of genes related to the toll-like receptor 4 (TLR-4)/nuclear factor-kappa B (NF-κB)/nod-like receptor protein 3 (NLRP3) inflammatory signaling pathway in the liver of HFD-fed rats was notably suppressed by SA. In terms of the gut microbiota, the LSA group showed a significantly higher fecal abundance of *Oscillospiraceae_UCG_005*, *Butyricicoccaceae_UCG_009* and *Colidextribacter* compared with the HFD group (*p* < 0.05). The rats in the HSA group had a higher abundance of *unclassified_Lachnospiraceae*, *Colidextribacter* and *Oscillibacter* compared with the HFD-associated gut community (*p* < 0.05). In addition, rats treated with SA showed a significant increase in fecal short chain fatty acids (SCFAs) levels and a decline in serum lipopolysaccharide (LPS) levels compared with the HFD group (*p* < 0.05). Moreover, the modulated bacteria and microbial metabolites were notably correlated with the amelioration of NAFLD-related indices and activation of the hepatic TLR4/NF-κB/NLRP3 pathway. In conclusion, SA prevented NAFLD and the potential mechanism was related to the modulation of the gut–liver axis.

## 1. Introduction

Non-alcoholic fatty liver disease (NAFLD) is defined as excessive fat deposition in the hepatocyte caused by liver-damaging factors instead of alcohol [1]. NAFLD is the predominant liver disorder worldwide in parallel with the epidemics of obesity and diabetes mellitus [2]. Nowadays, the prevalence of NAFLD is about 29.62% in the adults globally and 29.81% in China [3]. NAFLD can lead to increased cirrhosis and hepatic carcinoma risk [4] and is closely related to the progression of cardiovascular diseases [5], chronic kidney disease [6] and other metabolic diseases [7]. Currently, the pathogenesis of NAFLD has not been clearly elucidated. Increasing evidence indicates that the changes of gut microbiota and its metabolites potentially modulates NAFLD through the gut–liver axis [8]. Studies have shown that NAFLD patients are often accompanied by intestinal microbiota disorders [9].

The gut–liver axis is a crosstalk between the gut and liver through the portal vein [10], which causes the liver to be continuously exposed to gut-derived metabolites. For instance, a high-fat diet (HFD) could increase the intestinal abundance of lipopolysaccharide (LPS) producing bacteria [11]. These harmful metabolites provoke the damage of intestinal mucosal barriers and enhance intestinal permeability, leading the gut-derived LPS flowing into the liver and triggering chronic inflammation [12]. LPS is involved in the activation of toll-like receptor 4 (TLR4). Activated TLR-4 could further stimulate the expression of nuclear factor-kappa B (NF-κB) [13,14] and nod-like receptor protein 3 (NLRP3) inflammatory signal pathway related proteins in the liver, and ulteriorly initiate the secretion of pro-inflammatory cytokines, including interleukin 1β (IL-1β) [15]. Inversely, HFD could suppress the expansion of probiotics and reduce the fecal levels of short chain fatty acids (SCFAs), such as acetic acid, propionate and butyrate [16]. SCFAs, generated by fermentation of dietary fiber, have the effect of preventing inflammation [17]. For instance, supplementation of acetate displayed an effective decline in methionine-choline deficient (MCD)-induced macrophage aggregations and proinflammatory responses [18]. Sodium butyrate treatment ameliorated HFD-induced gut flora disorder and suppressed low-grade chronic inflammation in obese mice [19]. Hence, regulating intestinal flora and its metabolites may be critical in the prevention of chronic inflammation and the development of NAFLD.

Nowadays, there are no effective drugs available for NAFLD treatment while the therapeutic effects of some dietary fibers, such as resistant starch [20] and inulin [21], on amelioration of NAFLD have attracted the attention of researchers. Sodium alginate (SA), a water-soluble dietary fiber, is mainly obtained from brown seaweed [22]. Several studies have suggested that SA is beneficial for ameliorating NAFLD and they have proven the modulation effect of SA on intestinal flora and inflammation [23,24]. A study reported that SA improved hyperlipidemia by modulating gut microbiota composition [25]. Another study suggested SA improved MCD-induced hepatic inflammation by maintaining intestinal barrier function [26]. A recent study indicated that SA improved HFD diet-induced metabolic disorders were associated with changes in gut microbial structure in mice [27]. However, no study has systematically explored the roles of SA-regulated gut microbiota and related metabolites on the progression of NAFLD.

In the present study, we hypothesized that SA may ameliorate NAFLD by modulating the gut–liver axis. Therefore, the aim of this study was to investigate the anti-inflammatory effect of SA on HFD-fed rats and evaluate whether its potential benefits are related to the regulation of gut microbiota and its metabolites. The findings will provide further evidence for clarifying the effects of SA intervention on NAFLD.

## 2. Materials and Methods

### 2.1. Animal Experiment Design

This study was authorized by the laboratory animal welfare ethics committee of Qingdao University (20210520SD3620210923020). Male Sprague–Dawley rats (180–220 g) were obtained from Sipford Biotechnology Co., LTD (Beijing, China) and housed under specific conditions (temperature: 22–25 °C, humidity: 45–55% and 12 h- light/dark cycle). After acclimatization, the animals were randomly separated into 4 groups (*n* = 8 per group): (1) normal control group (NC) fed with a normal chow diet (Diet serial number: D12450H; 10% calories from fat) and gavaged with normal saline; (2) high-fat diet group (HFD) fed with a HFD (Diet serial number: D12451; 45% calories from fat) and gavaged with normal saline; (3) low dose sodium alginate group (LSA), fed with a HFD and gavaged with 50 mg/kg/d SA; (4) high dose sodium alginate group (HSA) fed with a HFD and gavaged with 150 mg/kg/d SA. The dosage of SA was determined according to previous the literature [24,28]. SA was provided by Bright moon seaweed group Co. Ltd. (Qingdao, China). Each rat was housed in a separate cage.

After 16 weeks of feeding, the rats were anesthetized and scarified followed 12 h fasting to collect blood and tissues. The blood was centrifugated at 2000× *g* and 4 °C for 10 min to obtain serum. Liver, epididymal and perirenal adipose tissues were weighted and stored for analysis. Cecal contents were collected from each rat aseptically for bacterial DNA sequencing. All samples were rapidly frozen in liquid nitrogen and stored at −80 °C.

### 2.2. Serum Biochemical and Inflammatory Markers Analysis

Serum concentrations including triglyceride (TG), total cholesterol (TC), high-density lipoprotein-cholesterol (HDL-C), low-density lipoprotein-cholesterol (LDL-C), alanine aminotransferase (ALT) and aspartate aminotransferase (AST) were detected using an automatic biochemical analyzer. Serum tumor necrosis factor (TNF-α) was measured using an ELISA kit (Bioscience, Inc., Thermo, Rancho Cucamonga, CA, USA). Serum lipopolysaccharide (LPS) was detected according to ELISA kit instructions (Jingmei, Yancheng, Jiangsu, China). Liver TG and TC were measured by commercial kits (Nanjing Jiancheng Bioengineering Institute, Nanjing, Jiangsu, China).

### 2.3. Histopathological Examination

The fresh liver tissues were washed with normal saline and fixed with 10% formaldehyde buffer for 24 h. Then, the tissue slices were dehydrated and embedded in paraffin to be cut into a thickness of 4 µm and stained with hematoxylin and eosin (H&E). The sections were observed on a light microscope (Olympus, Tokyo, Japan) (400×) for the degree of hepatic steatosis and inflammation. The NAFLD activity score (NAS) was determined by the following literature [29].

### 2.4. Western Blot Analysis

30 mg of liver tissues was homogenized in cold RIPA lysis buffer and then centrifuged to collected supernatant. The extracted total proteins were qualified by the BCA Protein Quantitation Kit (Yase Biotechnology, Shanghai, China). Equal amounts of proteins were added onto 10% SDS-PAGE and then transferred to PVDF membranes, which were blocked with 5% skim milk and subsequently incubated with the following primary antibodies overnight at 4 °C (1:1000, dilution): TLR-4 (Affinity, Changzhou Jiangsu, China. Lot#16c5074), NF-κB (Immunoway, Suzhou, Jiangsu, China. Lot#B0801), NLRP3 (Abcam Bioscience, New York, NJ, USA. Lot#GR3369-573), caspase-1 (Affinity, Changzhou, Jiangsu, China. Lot#23m5315), IL-1β (Abcam Bioscience, New York, NJ, USA. Lot#GR3354222-10) and β-actin (Abcam Bioscience, New York, NJ, USA. Lot#GR3249122-24). The primary antibody reaction membrane was washed with TBST and incubated with a secondary antibody (Absin, Shang Hai, China. Lot#AS010 and Lot#AS006) (1:7500, dilution) for 1.5 h. The β-actin was utilized as a control. The protein bands were observed by an enhanced chemiluminescence (ECL) localization reagent and quantified by Tanon GIS analysis.

### 2.5. Real-Time Quantitative PCR Analysis

TRIzol reagent and a NanoDrop spectrophotometer (NanoDrop Technologies, Wilmington, DE, USA) were utilized to extract total RNA from the liver tissues and evaluate the amount and purity of RNA, respectively. Then, about 1 μg RNA was reversely transcribed into cDNA using a PrimeScript RT Master Mix (TaKaRa, Beijing, China). The real-time quantitative PCR was carried out using SYBR Premix Ex Taq fluorescent quantitative PCR (Eppendorf, Framingham, MA, USA). The conditions for qPCR were as follows: initial denaturation at 95 °C for 30 s, followed by 40 cycles at 95 °C for 5 s and 60 °C for 32 s. The primers are shown in Table S1.

### 2.6. Sequencing of Microbiota and Analysis

Microbial sequencing of rats’ cecal contents were completed by Beijing Biomarker Technologies Co., Ltd. (Beijing, China). Briefly, bacterial DNA was extracted from cecal contents samples with a QIAamp Fast DNA Stool Mini Kit (Qiagen, Germany). PCR amplification was conducted with barcoded specific bacterial primers targeting the variable region 3–4 (V3–V4) of the 16S rRNA gene: forward primer 338F: 5′- ACTCCTACGGGAGGCAGCA-3′ and reverse primer 806R: 5′- GGACTACHVGGGTWTCTAAT-3′. Construction of sequencing libraries and paired end sequencing were performed on an Illumina NovaSeq6000 platform according to standard protocols. Paired-end reads were merged using FLASH v1.2.7. Taxonomy was assigned to all OTUs by searching against the Silva databases (Release128) using QIIME software.

The alpha-diversity and beta-diversity of intestinal flora were estimated online (https://www.microbiomeanalyst.ca./) accessed on 10 August 2022. Correlation analysis was executed on the basis of Spearman’s rank correlation test, and a heat map was performed with HemI 1.0.

### 2.7. Measurement of Short Chain Fatty Acids in Cecal Contents

Samples of 100 mg of cecum content were mixed with 0.27 mL of purified water, 0.5 mL of acetone, 0.05 mL of 50% sulfuric acid, 0.5 mL of methyl tert-butyl ether and 200 mg sodium chloride and allowed to stay for 5 min. Then, the mixture was centrifuged for 10 min (12000× *g*, 4 °C) to acquire the supernatant. The levels of SCFAs were detected by GC/MS (Agilent 7890-7250 GC/MSD, Santa Clara, CA, USA).

### 2.8. Statistical Analysis

Data were expressed as mean ± standard deviation (SD). The one-way analysis of variance (ANOVA) followed by LSD post-hoc test was applied to determine the differences for all variables among the groups. IBM Social Science (SPSS) version 26.0 and GraphPad Prim 8.0 were used for analysis and drawing, respectively. The significance level was *p* < 0.05.

## 3. Results

### 3.1. SA Supplementation Reduced Body Weight

**Figure 1:**
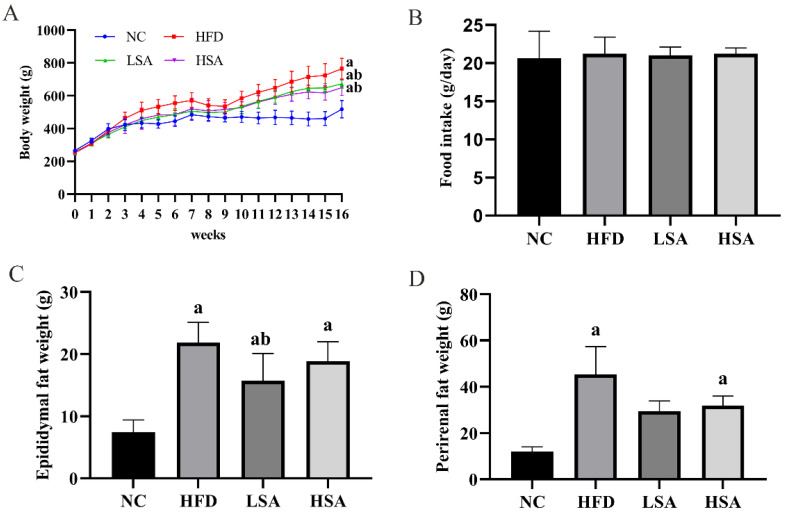
SA supplementation reduced body weight. (**A**) Weight change during the 16-week experiment; (**B**) food intake; (**C**) epididymal fat weight; (**D**) perirenal fat weight. *n* = 8 each group. ^a^ *p* < 0.05, compared to the NC group; ^b^ *p* < 0.05, compared to the HFD group.

As shown in Figure 1A, the weight of rats in the HFD group was higher than that in the NC group after 16 weeks of feeding. Compared with the HFD group, the final body weight of LSA group and HSA group decreased by 17.4% and 21.6%, respectively (*p* < 0.05 for both). No significant differences in food intake were observed among the four groups (*p* > 0.05, Figure 1B). As shown in Figure 1C,D, the weight of epididymal and perirenal fat in the HFD group were significantly higher than those in the NC group (*p* < 0.05) and the LSA group significantly decreased the weight of epididymal fat (*p* < 0.05).

### 3.2. SA Supplementation Ameliorated NAFLD-Related Parameters

**Figure 2:**
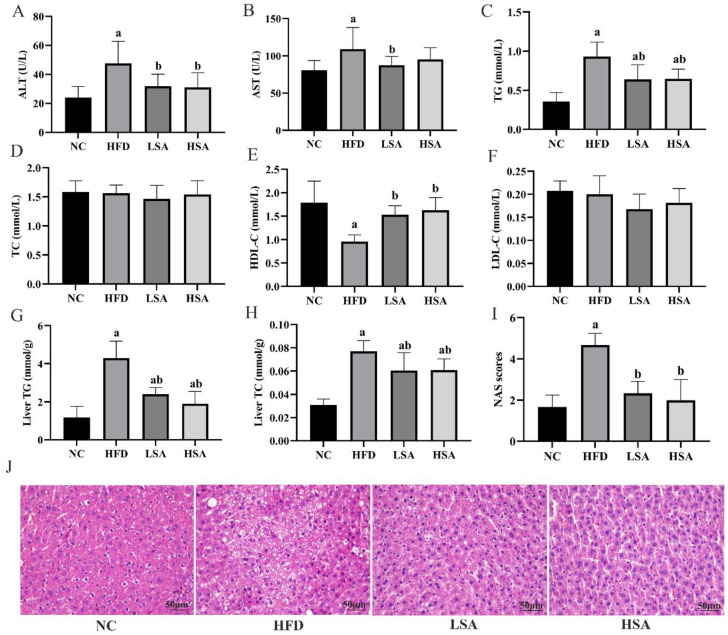
SA supplementation ameliorated NAFLD-related parameters. (**A**) Serum ALT; (**B**) serum AST; (**C**) serum TG; (**D**) serum TC; (**E**) serum HDL-C; (**F**) serum LDL-C; (**G**) liver triglyceride (liver TG); (**H**) liver total cholesterol (liver TC); *n* = 8 each group for (**A**–**H**); (**I**) NAS scores; (**J**) liver tissues with H&E staining (magnification, ×400; scale bars, 50 µm; *n* = 3 each group). ^a^ *p* < 0.05, compared to the NC group; ^b^ *p* < 0.05, compared to the HFD group.

As shown in Figure 2, the contents of serum ALT and AST in the HFD group were higher than those in the NC group (*p* < 0.05). The LSA and HSA groups had lower serum ALT levels compared to NAFLD rats (*p* < 0.05). Moreover, the levels of TG and TC in both the serum and liver were significantly elevated after the administration of HFD (*p* < 0.05). SA-treated rats exhibited significantly lower levels of serum TG and hepatic TG and TC concentrations than the HFD group (*p* < 0.05). Moreover, the HFD group showed a significant reduction in the serum HDL-C levels compared to the NC group (*p* < 0.05). SA treatment significantly enhanced serum HDL-C levels (*p* < 0.05). No changes were observed in serum TC and LDL-C levels among the groups. Interestingly, the ratio of TC/HDL-C in the rats in the HFD group was significantly higher than that of rats in the NC group (*p* < 0.05). Both LSA and HSA groups had a reduced TC/HDL-C ratio compared with the HFD group (*p* < 0.05, Figure S1).

Micrographs of HE-stained hepatic tissues (Figure 2J) showed that, compared with rats in NC group, the livers of rats in the HFD group exhibited about 60% steatosis characterized by swelling hepatocytes and fat vacuoles accumulation accompanying inflammatory cell infiltration. SA intervention efficiently attenuated the degree of hepatocyte steatosis, decreased the number of fat droplet hepatic lobules and infiltrated the inflammatory cells. The NAS score that considered steatosis and inflammation was higher (*p* < 0.05) in the HFD group than in the NC group. The NAS score was dramatically decreased after SA treatment in comparison with the HFD group. (*p* < 0.05, Figure 2I).

### 3.3. SA Supplementation Inhibited Hepatic Inflammation

**Figure 3:**
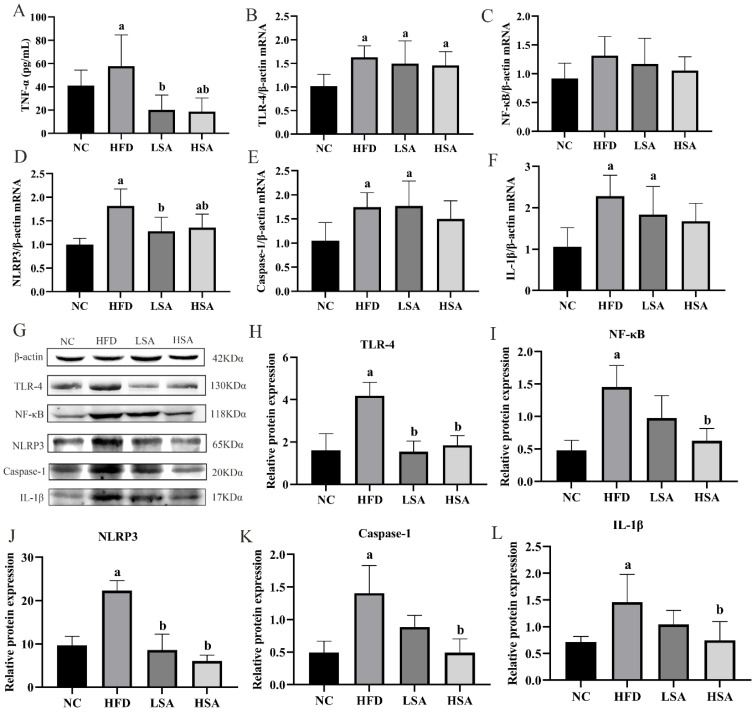
SA supplementation inhibited hepatic inflammation. (**A**) Serum TNF-α, *n* = 8 each group; (**B**–**F**) relative mRNA expression of TLR-4, NF-κB, NLRP3, caspase-1 and IL-1β, *n* = 6 each group; (**G**) western blot assay of proteins in liver, *n* = 3 each group; (**H**) quantitative analyses of the TLR-4 protein; (**I**) quantitative analyses of the NF-κB protein; (**J**) quantitative analyses of the NLRP3 protein; (**K**) quantitative analyses of the caspase-1 protein; (**L**) quantitative analyses of the IL-1β protein. ^a^ *p* < 0.05, compared to the NC group; ^b^ *p* < 0.05, compared to the HFD group.

HFD-induced NAFLD is often accompanied by chronic inflammation. As shown in Figure 3A, elevated serum levels of TNF-α in the HFD-fed rats were ameliorated by SA supplementation (*p* < 0.05). As shown in Figure 3B–F, the mRNA levels of TLR-4, NLRP3, caspase-1 and IL-1β were elevated (*p* < 0.05) and NF-κB mRNA in the liver had an increased trend in the HFD group in comparison with the NC group. The mRNA of NLRP3 were dramatically downregulated in the SA-treated groups compared to the HFD-fed rats (*p* < 0.05). Moreover, the protein expression of TLR-4, NF-κB, NLRP3, caspase-1 and IL-1β were notably upregulated in the HFD group and inhibited in the SA intervened groups (*p* < 0.05).

### 3.4. SA Supplementation Altered Overall Gut Microbial Structure

**Figure 4:**
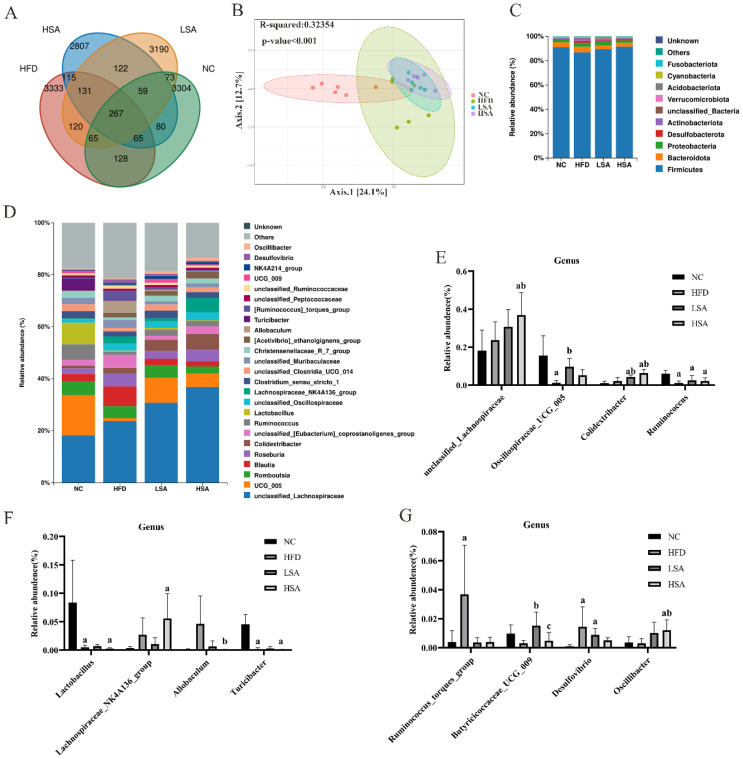
SA supplementation altered overall gut microbial structure. (**A**) Venn diagram, the numbers indicated sequencing reads; (**B**) principal coordinates analysis; (**C**) the microbial distributions at the phylum level; (**D**) the microbial distributions at the genus level; (**E−G**) relative abundance of bacteria at the genus level. *n* = 5−6 each group. ^a^ *p* < 0.05, compared to the NC group; ^b^ *p* < 0.05, compared to the HFD group; ^c^ *p* < 0.05, compared to the LSA group.

A total of 12634 fecal microbiota sequencing reads were analyzed, of which 3304, 3333, 3190 and 2807 reads were on average in the NC, HFD, LSA and HSA groups, respectively (Figure 4A). As shown in Figure 4B, the PCoA plot showed that the NC, HFD, LSA and HSA groups were clearly separated (*p* < 0.001). The ACE and Chao 1 indexes reflected the community richness while the Shannon and Simpson indexes indicted the community diversity. There was no change in four indexes among the NC, HFD and SA groups (Figures S2 and S3). Figure 4C shows the abundance of phyla levels in the bacterial communities. Compared to the HFD group, the level of *Actinobacteriota* was lower in the HSA group (*p* < 0.05). No significant differences were observed on the other phylum among the groups. At the genus level (Figure 4D–G), the HFD group displayed a higher abundance of *Desulfovibrio* (LPS-producing bacteria) and *Ruminococcus_torques_group* and lower abundance of SCFA-producing bacteria (*Oscillospiraceae_UCG_005*), *Ruminococcus*, *Lactobacillus* and *Turicibacter* in comparison with the NC group (*p* < 0.05 for all). In addition, the gut community of LSA rats showed a significantly higher abundance of *Oscillospiraceae_UCG_005*, *Butyricicoccaceae_UCG_009* and *Colidextribacter* compared with the HFD-associated gut community (*p* < 0.05). The gut community of HSA rats showed a significantly higher abundance of *unclassified_Lachnospiraceae*, *Colidextribacter*, *Oscillibacter* and lower levels of *Allobaculum* compared with the HFD-associated gut community (*p* < 0.05).

### 3.5. SA Supplementation Affected the Gut Microbiota-Derived Metabolites

**Figure 5:**
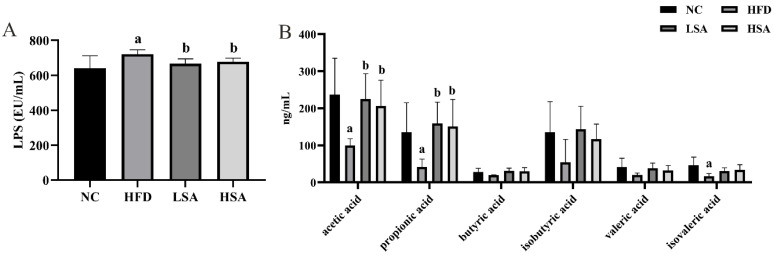
SA supplementation affected the gut microbial-derived metabolites. (**A**) Serum LPS; *n* = 8 each group; (**B**) fecal levels of SCFAs, *n* = 6 each group. ^a^ *p* < 0.05, compared to the NC group; ^b^ *p* < 0.05, compared to the HFD group.

Gut microbiota metabolites (SCFAs and LPS) were determined, and the experimental data are represented in Figure 5. The serum levels of LPS in the HFD group were higher than those in the NC group (*p* < 0.05). The increased serum LPS in the HFD group was decreased with SA intervention (*p* < 0.05). Administration of HFD reduced the fecal concentrations of acetic acid, propionic acid and isovaleric acid by 57.9%, 69.4% and 64.0%, respectively (*p* < 0.05). SA intervention dramatically increased the acetic acid and propionic acid levels in the feces (*p* < 0.05).

### 3.6. Correlation Analysis

**Figure 6:**
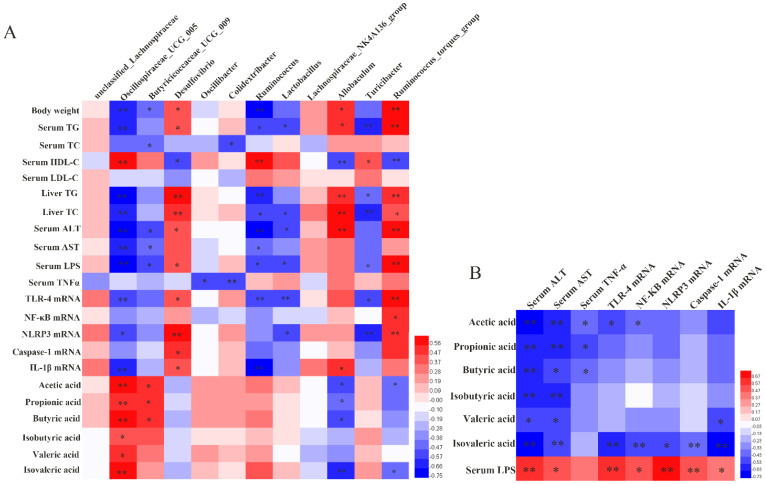
Correlation Analysis. (**A**) The heat map shows the Spearman correlation coefficient among the intestinal differential bacteria, lipid metabolism index, LPS, SCFAs and inflammation indicators; (**B**) the heat map shows the Spearman correlation coefficient between metabolites of gut microbiota (LPS and SCFAs) and inflammation indicators. * *p* < 0.05 and ** *p* < 0.01.

To determine whether gut microbiota and its metabolites remodeled by SA demonstrate liver-protective effects, Spearman correlation analysis was performed to identify the relevance among gut flora and its metabolites with NAFLD-related metabolic indicators. As exhibited in Figure 6A, the abundance of *Oscillospiraceae_UCG_005* and *Butyricicoccaceae_UCG_009* showed negative correlations with most of the NAFLD-related indices and positive correlations with SCFAs (*p* < 0.05). *Colidextribacter* and *Oscillibacter* displayed negative correlations with serum TNF-α (*p* < 0.05). Moreover, these probiotics, such as *Ruminococcus*, *Lactobacillus* and *Turicibacter*, were inversely related to LPS, lipids and inflammatory indicators (*p* < 0.05). Moreover, the relative abundance of *Desulfovibrio*, *Allobaculum* and *Ruminococcus_torques_group* exhibited a strong positive correlation with most of the NAFLD- and inflammation-related indicators (*p* < 0.05). *Allobaculum* and *Ruminococcus_torques_group* were also negatively correlated with SCFAs (*p* < 0.05). No correlations were noticed among *unclassified_Lachnospiraceae* and *Lachnospiraceae_NK4A136_group* with all of the NAFLD-related parameters.

As shown in Figure 6B, SCFAs were negatively correlated, while LPS was positively correlated with most of the inflammatory biomarkers (*p* < 0.05).

## 4. Discussion

In the current study, a NAFLD rat model was established by 16 weeks of HFD feeding, which was characterized by an increased accumulation of fat in the liver, decreased liver function, and elevated serum levels of lipids and inflammatory factors. Notably, all of these damages were reversed by SA supplementation. Of note, we first proved that the ameliorative effect of SA on NAFLD was related to the regulation of the gut–liver axis.

SA, a water-soluble polysaccharide isolated from brown seaweed, has been identified to display various biological activities, including anti-hyperlipidemia, anti-inflammation [26] and anti-obesity [27]. In the present study, both low-dose (50 mg/kg/d) and high-dose (150 mg/kg/d) SA supplementation remarkably inhibited hepatic lipids accumulation and decreased serum levels of ALT and AST in the HFD-fed rats, supporting the notable protective effect of SA on NAFLD [24,25]. NAFLD is associated with dyslipidemia and will increase the risk of cardiovascular diseases, such as atherosclerosis [30]. Herein, HFD induced elevated serum levels of TG and decreased those of HDL-C were dramatically attenuated by SA, which is in line with previous reports [31]. Intriguingly, SA treatment also reduced the high ratio of TC/HDL-C, which is a well-accepted predictor of atherosclerotic disease [32]. Collectively, these results indicate the potential protective effects of SA on lipid metabolic disorders and cardiovascular diseases. Moreover, SA notably suppressed body weight gain and accumulation of visceral body fat in the HFD-fed rats, which is in agreement with previous studies [27]. As is well known, obesity is a primary causal factor for NAFLD. Thus, we suppose the preventive effect of SA on NAFLD might be at least partially related to the amelioration of obesity.

NAFLD is recognized as a hepatic chronic inflammatory status which plays a vital role in its development. Our data displayed that the protein expression levels of inflammation-related genes, such as TLR-4, NF-κB, NLRP3, caspase-1 and IL-1β, were elevated in NAFLD rats, indicating the activation of the TLR-4/NF-κB/NLRP3 inflammatory pathway. TLR-4 is a critical regulator of the inflammatory response during HFD-diet-induced hepatic injury. Activation of TLR-4 regulates NF-κB and further initiates NLRP3 inflammasome. The role of NLRP3 in liver diseases has attracted increased attention and has been considered as a potential new therapeutic target for NAFLD [33,34]. NLRP3 inflammasome is a cytoplasmic multiprotein complex composed of NLRP3, ASC and caspase-1. NLRP3 binding to ASC causes the activation of caspase-1 which promotes the maturation of IL-1β from pro IL-1β [35]. Pro-inflammatory cytokines IL-1β induce hepatic steatosis, aggravate the inflammatory reaction, and further promote the occurrence and development of NAFLD [36]. In our study, we found that SA significantly downregulated the protein levels of TLR-4, NF-κB, NLRP3, caspase-1 and IL-1β in the livers of NAFLD rats, indicating the inhibition of the TLR-4/NF-κB/NLRP3 pathway. Consistent with our findings, some studies have also reported the ameliorative effect of SA on inflammation. Huang et al. found that SA decreased gut inflammation by downregulating the expression of TLR-4 in immunosuppressed BALB/c mice [37]. Kawauchi et al. found SA administration improved hepatic inflammation by decreasing the mRNA expression of TNF-α in MCD-induced NASH mice [26]. Miyazaki T et al. found that SA supplementation reduced the mRNA expression levels of IL-1β and TNF-α in the livers of obese mice [38]. Collectively, we proved that the improvement effect of SA on NAFLD was related to the suppression of the TLR-4/NF-κB/NLRP3 pathway in the liver.

Intestinal flora and its metabolites account for important roles in the pathogenesis of NAFLD, such as triggering inflammation [39,40]. In the gut of patients and animals with NAFLD, the abundance of some pernicious bacteria was increased, such as *Ruminococcus_torques_group* and LPS-containing *Desulfovibrio* [41,42]. HFD can contribute to the impairment of gut mucosal barriers’ integrity and permeability, increasing serum LPS levels [43]. As is well known, the LPS derived from gut pathogenic bacteria is a natural TLR ligand [44] and an important promoter of hepatic chronic inflammation [45]. Abnormal translocated LPS binds to the TLR-4 of the liver to activate the TLR-4/NF-κB/NLRP3 pathway, and further expands the inflammatory response [46,47,48,49]. On the other hand, *Oscillospiraceae_UCG_005* [50], *Butyricicoccaceae_UCG_009*, *Ruminococcus* [51], *Lactobacillus* [52] and *Oscillibacter* [53] are SCFA-producing microbiota. SCFAs, including acetic acid, propionate, butyrate, etc., are beneficial for preventing chronic inflammation. Acetic acid alleviated the inflammatory response and liver injury by downregulating the activity of the TLR4 signaling pathway in septic mice [54]. Propionate reduced the LPS-induced inflammatory response in mouse endothelial cells and inhibited the activation of the TLR-4 protein on the surface of alveolar macrophages [55]. Butyrate also suppressed high-fat diet-induced systematic inflammation [56]. Herein, the abundance of *Desulfovibrio* and *Ruminococcus_torques_group* was relatively decreased in the SA groups. Accordingly, the serum levels of LPS were also markedly reduced after SA treatment. In addition, we found that the abundance of *Oscillospiraceae_UCG_005*, *Butyricicoccaceae_UCG_009*, *Oscillibacter*, and the fecal levels of SCFAs in the SA treatment rats were significantly enhanced compared with the NAFLD rats. To further verify whether SA inhibited hepatic inflammation by regulating gut bacteria and its metabolites, we performed a Spearman correlation analysis. The SCFAs-producing bacteria and the levels of SCFAs in the feces were negatively related to inflammation indicators, such as ALT and mRNA levels of TLR-4 and NLRP3. Positive correlations were revealed among *Desulfovibrio* and *Ruminococcus_torques_group* with LPS and inflammatory markers. *Colidextribacter* [57] and *Turicibacter* [58] are anti-inflammatory probiotics. In the present study, *Colidextribacter* was also enriched in the feces by SA and *Turicibacter* was negatively correlated with hepatic inflammation. Thus, we surmise that the protective effect of SA on NAFLD might be explained by the remodeling of gut microbiota composition and the production of LPS and SCFAs, counteracting hepatic TLR-4/NF-κB/NLRP3 pathway-mediated inflammation.

## 5. Conclusions

**Figure 7:**
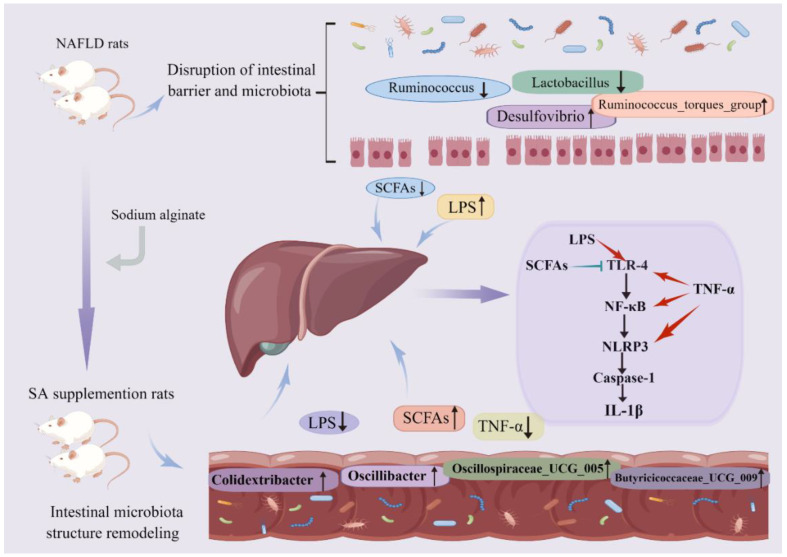
SA-alleviated inflammation by modulating the gut–liver axis in HFD-fed rats. The black upward arrows represent increase; the black downward arrows represent decrease; the red arrows represent promotion.

Our results proved that SA ameliorates NAFLD via modulating gut microbiota and its metabolites to suppress the TLR4/NF-κB/NLRP3 inflammatory pathway in the livers of rats (Figure 7). In addition, metabonomic approaches are needed to identify the other microbial metabolites which are involved in the preventive effects of SA on NAFLD in the future. The current work has provided a novel insight for the preventive effects and mechanisms of SA on NAFLD in vivo and has provided guidance for the prevention and clinical remedy of NAFLD.

## Data Availability

Not applicable.

## Supplementary Materials

The following supporting information can be downloaded at: https://www.mdpi.com/article/10.3390/nu14224846/s1, Table S1: Primers used during the real-time PCR assay. Figure S1: The ratio of TC/HDL-C. Figures S2 and S3: The alpha-diversity.
