# Supplementary material for: Sodium Alginate Prevents Non-Alcoholic Fatty Liver Disease by Modulating the Gut–Liver Axis in High-Fat Diet-Fed Rats
Source: Nutrients. 2022 Nov 16;14(22):4846. doi: 10.3390/nu14224846 (PMC9697635; doi:10.3390/nu14224846)
Supplement: Supplementary file 1 [file nutrients-14-04846-s001.zip › nutrients-1972476-supplementary.pdf]

Table S1. Primers used during the real-time PCR assay

Gene	Forward/reverse primer（5′-3′）
TLR-4	F: 5′- CCGCTCTGGCATCATCTTCA -3′
	R: 5′- CCCACTCGAGGTAGGTGTTTCTG -3′
NF-κB	F: 5′- GATGGGACGACACCTCTACACATA -3′
	R: 5′- CCCAAGAGTCGTCCAGGTCA -3′
Caspase-1	F: 5′- ACTCGTACACGTCTTGCCCTCA -3′
	R: 5′- CTGGGCAGGCAGCAAATTC -3′
NLRP3	F: 5′- CTGAAGCATCTGCTCTGCAACC -3′
	R: 5′- AACCAATGCGAGATCCTGACAAC -3′
IL-1β	F: 5′- CCCTGAACTCAACTGTGAAATAGCA -3′
	R: 5′- CCCAAGTCAAGGGCTTGGAA -3′
β-actin	F: 5′- GGAGATTACTGCCCTGGCTCCTA -3′
	R: 5′- GACTCATCGTACTCCTGCTTGCTG -3′

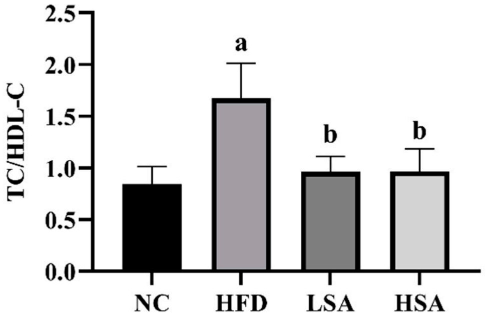

Figure S1: The ratio of TC/HDL-C. n = 8 each group. $^{a}P < 0.05$, compared to the NC group; $^{b}P < 0.05$, compared to the HFD group.

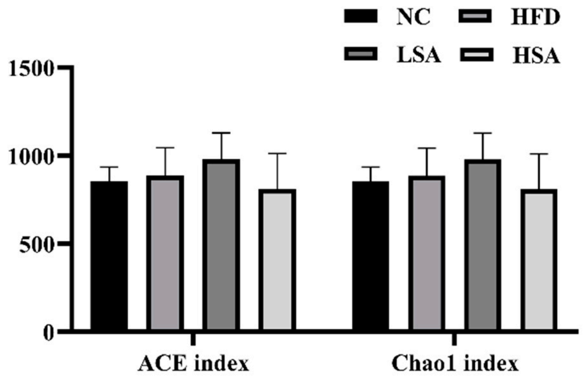

Figure S2: The alpha-diversity. n = 5-6 each group. $^{a}P < 0.05$, compared to the NC group; $^{b}P < 0.05$, compared to the HFD group.

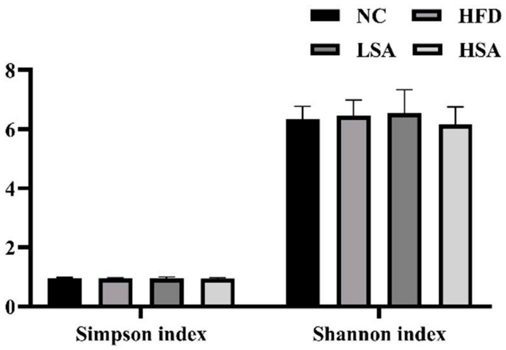

Figure S3：The alpha-diversity. n = 5-6 each group. $^{a}P < 0.05$, compared to the NC group; $^{b}P < 0.05$, compared to the HFD group.
